# Comparison of histopathology to gene expression profiling for the diagnosis of metastatic cancer

**DOI:** 10.1186/1746-1596-7-110

**Published:** 2012-08-21

**Authors:** Anand Kulkarni, Raji Pillai, Ashley M Ezekiel, W David Henner, Charles R Handorf

**Affiliations:** 1 University of Tennessee Health Science Center, 930 Madison, 5th Floor, Memphis, TN 38163, USA; 2 Pathwork Diagnostics, 595 Penobscot Drive, Redwood City, CA 94063, USA

**Keywords:** Metastatic cancer, Poorly differentiated cancer, Tumors of uncertain origin, Cancer of unknown primary, Immunohistochemistry, Gene expression profiling, Digital pathology

## Abstract

**Background:**

Determining the primary site of metastatic cancer with confidence can be challenging. Pathologists commonly use a battery of immunohistochemical (IHC) stains to determine the primary site. Gene expression profiling (GEP) has found increasing use, particularly in the most difficult cases. In this pilot study, a direct comparison between GEP and IHC-guided methods was performed.

**Methods:**

Ten archived formalin-fixed paraffin embedded metastatic tumor samples for which the primary site had been clinically determined were selected. Five pathologists who were blinded to the diagnosis were asked to determine the primary site using IHC and other stains selected from a panel of 84 stains. Each pathologist was provided patient sex, biopsy site and gross sample description only. Slides were digitized using ScanScope®XT at 0.25 μm/pixel. Each evaluating pathologist was allowed to provide a diagnosis in three stages: initial (after reviewing the H&E image), intermediate (after reviewing images from the first batch of stains) and final diagnosis (after the second batch of stains if requested). GEP was performed using the only FDA-cleared test for this intended use, the Pathwork Tissue of Origin Test. No sample information was provided for GEP testing except for patient sex. Results were reported as the tumor tissue type with the highest similarity score.

**Results:**

In this feasibility study, GEP determined the correct primary site in 9 of the 10 cases (90%), compared to the IHC-guided method which determined the correct primary site for 32 of 50 case evaluations (average 64%, range 50% to 80%). The five pathologists directing the IHC-guided method ordered an average of 8.8 stains per case (range 1 to 18). GEP required an average of 3 slides per case (range 1 to 4).

**Conclusions:**

Results of the pilot study suggest that GEP provides correct primary site identification in a higher percentage of metastatic cases than IHC-guided methods, and uses less tissue. A larger comparative effectiveness study using this study design is needed to confirm the results.

**Virtual slides:**

The virtual slide(s) for this article can be found here: http://www.diagnosticpathology.diagnomx.eu/vs/1749854104745508

## Background

Determining the primary site of metastatic cancer with confidence can be challenging; for 3–5% of cancer cases there is no clinically evident primary site [1-7]. Pathologists commonly use panels of immunohistochemical (IHC) and histochemical (HC) stains for diagnosis. A judicious use of lineage and organ-specific tissue markers is required to diagnose the primary site of metastatic cancer, as markers vary in levels of sensitivity and specificity. [6]

In some cases, however, the primary site cannot be identified with certainty using conventional IHC evaluation. In a review and meta-analysis of published studies of IHC accuracy that were adequately blinded, four studies representing a total of 308 tumor samples reported average accuracy of 67% on metastatic samples [8].

In recent years, gene expression profiling (GEP) tests and tests using microRNA markers have been developed to aid in the diagnosis of difficult-to-diagnose tumors [9-11]. These tests use formalin-fixed paraffin-embedded (FFPE) tissue and either microarrays or real-time polymerase chain reaction technologies to measure levels of multiple markers followed by application of an algorithm to predict the most likely primary site for a particular sample.

Recent reports discuss the need to coordinate the use of available methods of identifying primary site to optimize patient care [12,13]. However, to date no direct comparison of the accuracy of the GEP and IHC-based methods has been published; more data regarding comparative effectiveness of the gene expression- and IHC-based approaches are needed to fully understand the appropriate use of these two methods.

One of the GEP tests for primary site identification is the Tissue of Origin Test (Pathwork Diagnostics, Redwood City, California) [14-17]. We conducted a pilot study using 10 cases to compare accuracy of IHC-based diagnoses of primary site with diagnoses rendered by the Tissue of Origin Test. We also compared the amount of tissue used by each method, as preserving tissue is critical if additional diagnostic analyses are needed.

A web interface was developed which linked to a digital microscopy database to facilitate and standardize immunohistochemical evaluation by evaluating pathologists. Histology slides were digitized and the images were provided to evaluating pathologists for histopathological evaluation. Using digital pathology and secure web access provided a controlled means to allow access to only the stains requested by each pathologist and maintained the blinding of the pathologist.

The methods being compared have inherent differences that can confound a comparison – GEP is objective, independent of clinical history, based on algorithmic applications to a dataset, and is a one-time test, whereas histopathologic evaluation is subjective, reliant on clinical history and often performed in consultation with other pathologists [18]. Stains are typically ordered in batches, and cost considerations may influence how many IHC stains a pathologist orders. We attempted to eliminate such confounding sources of variation. We placed no limit on the number of stains that could be ordered, and required individual pathologist assessments through a secure web interface that prevented access to evaluations being conducted by the other participating pathologists. Both GEP and histopathology were conducted on the same tissue block and the same restricted information of patient gender and minimal gross description of the specimen was made available for both methods.

This publication describes the novel methods used to conduct the study, results of the pilot project, and provides information needed for conducting a more comprehensive study.

## Methods

**Figure 1:**
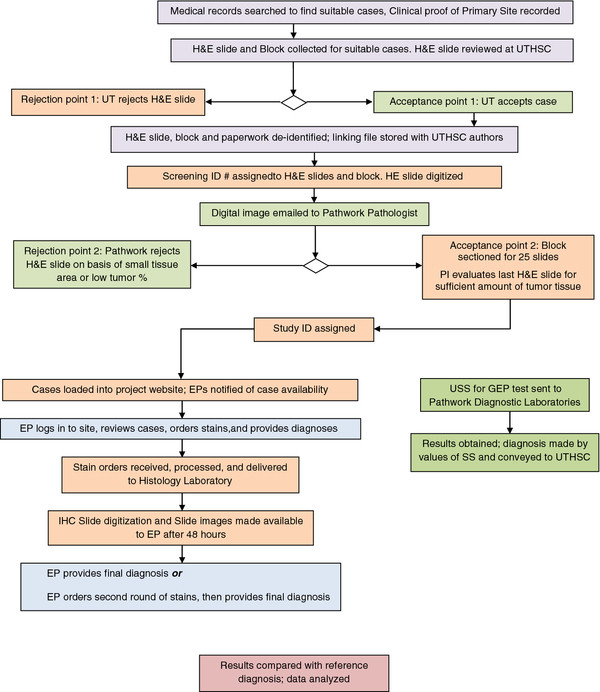
**Project workflow.** A summary of the project workflow is provided starting from the initial screening of the tissue specimen and the associated clinical information. Once qualified, the blinded specimens were processed for analysis by evaluating pathologists as well as GEP. The results from each approach were compared after processing was complete.

Archived human formalin-fixed paraffin-embedded (FFPE) specimens (blocks) containing metastatic tumors with a clinically established primary site were used. Samples were coded and both the evaluating pathologists and the laboratory performing the GEP at Pathwork Diagnostics were blinded to the primary site. The study was conducted under an Institutional Review Board-approved protocol. The overall study design and workflow is illustrated in Figure 1. Medical records from 2007 to 2011 were reviewed from two hospitals (The Regional Medical Center at Memphis, and Methodist University Hospital, Memphis, TN) to select cases with biopsy proven metastatic cancer chosen by the Principal Investigator to resemble cases on which GEP may be used in clinical practice, i.e. where the diagnosis was not always obvious upon morphology review.

### Specimen inclusion criteria

The selection criteria were as follows. (i) The sample represented a FFPE metastatic tumor with a known primary site as determined by review of the medical records. The determination of primary site was based on all available clinical information, but was not accepted when the diagnosis was made exclusively using immunohistochemistry (IHC) or special stains. (ii) All tumor samples were selected from a panel of 15 tumor tissue types covered by the Tissue of Origin Test. These types include: bladder (BL), breast (BR), colorectal (CO), gastric (GA), testicular germ cell (GC), kidney (KI), hepatocellular (LI), non-small cell lung (LU), non-Hodgkin’s lymphoma (LY), melanoma (ME), ovarian (OV), pancreas (PA), prostate (PR), thyroid (TH) and sarcoma (SC). (iii) The FFPE tissues submitted on unstained slides or paraffin blocks were checked for adequacy of tissue required for the study. Samples had to be sufficient to produce at least 25 5-μm-thick (+/− 1 μm) sections: (a) the first and last sections for staining with hematoxylin and eosin (H&E) stain, (b) eight unstained slides (USS) containing no less than 1 mm^2^ of tumor tissue for GEP, and (c) at least 15 unstained slides for IHC staining and analysis. (iv) H&E stained slides were evaluated by a board certified pathologist to verify tumor content. All specimens were estimated to contain ≥ 60% non-necrotic tumor tissue (tumor and stroma) in the first and last H&E stained slide and (v) found to be consistent with the reported histology on quality review by a board-certified pathologist. If the number of stains ordered exceeded the number of slides that were cut initially, additional slides were prepared from the block, and the last slide was stained with H&E to verify that at least 60% tumor content remained.

### Staining procedure and quality control

All stains were performed in a CLIA-certified laboratory by a histotechnologist with more than 20 years of IHC staining experience. All IHC stains were performed on a Ventana Benchmark ® LT automated immunohistochemical stainer. Relevant controls were included with each batch of stains. Prior to study initiation, a panel of 84 stains was agreed upon by all investigators, and made available for the study. This included 73 IHC stains and 11 histochemical stains (Table 1). Evaluating pathologists were free to order as many stains as desired from the list of stains in two rounds of requests.

**Table 1:** Pre-established list of immunohistochemical (IHC) and histochemical (HC) stains made available to evaluating pathologists

**No.**	**IHC Stain**	**No.**	**IHC Stain**	**No.**	**IHC Stain**	**No.**	**HC Stain**
1	Actin, muscle specific	26	CK17	51	Myogenin	1	Alcian blue- PAS
2	AE1/AE3	27	CK19	52	Napsin A	2	Argentaffin
3	AFP	28	CK5/6	53	NSE	3	Argyrophil
4	hCG	29	Pancytokeratin	54	OCT-4	4	Colloidal iron stain
5	CA-125	30	Desmin	55	P504S	5	Elastic, Verhoeff’s
6	Calcitonin	31	E-cadherin	56	p53	6	Mucicarmine
7	Caldesmon	32	EMA	57	p63	7	PAS
8	Calretinin	33	Estrogen Receptor	58	PAX-2	8	PASD
9	CAM 5.2	34	GCDFP-15	59	PAX5	9	PTAH
10	CD10	35	GFAP	60	PAX-8	10	Reticulum, Gomori’s
11	CD117	36	Glypican-3	61	PLAP	11	Trichrome, Masson’s
12	CD138	37	Hep-Par1	62	Progesterone Receptor		
13	CD20	38	HMB 45	63	Prostate Specific Antigen		
14	CD3	39	HMW Keratin	64	RCC		
15	CD30	40	HNF-1	65	S-100		
16	CD34	41	Inhibin	66	Synaptophysin		
17	CD43	42	CK, Oscar	67	Thrombomodulin		
18	CD45RO	43	Leucocyte Common Antigen	68	Thyroglobulin		
19	CD56	44	Mammaglobin	69	TTF1		
20	CD99	45	Melan-A	70	Uroplakin		
21	CDX2	46	MOC-31	71	Villin		
22	CEA-polyclonal	47	MUC 1	72	Vimentin		
23	Chromogranin	48	MUC 2	73	WT-1		
24	CK 20	49	MUC5AC				
25	CK 7	50	Myeloperoxidase				

### Digitizing stained slides

All H&E slides and IHC stained slides were digitized using a Whole Slide Imaging (WSI) system (ScanScope®XT, Aperio® Technologies, Inc., San Diego, CA). All slides were scanned at 0.25 μm/pixel resolution, and the images saved in the password-protected database (Spectrum version 10.2.2.2314) provided by Aperio® on a web-accessible server. All digitized images were reviewed by a board-certified pathologist for quality assurance.

### Selection of evaluating pathologists

Five board-certified pathologists with a wide range of experience (3 to 30+ years post pathology training) and from a diverse set of institutions (academic centers, community practice, and pathology reference laboratory) were selected for evaluating the cases. All evaluating pathologists confirmed previous use of digital pathology.

### Web interface

A web-accessible user interface was designed (OneTera LLC, San Francisco, CA) for evaluating pathologists to access the images in the Spectrum database, order stains as needed, and record diagnoses and associated confidence levels. This information was collected at the following points: a) after review of the H&E slide alone b) after review of the first batch of stains, and c) after review of the second batch of stains. Each evaluating pathologist (EP) had secure password-protected access to the interface. All 10 cases were evaluated by each EP. One H&E image was provided to all EPs as the starting point for each case. Each pathologist had access only to IHC stains that they had ordered; if a stain was ordered by more than one pathologist, requestors were provided with a copy of the same digital image. This added control and reduced variability in interpretation that might be attributed to the staining procedure or tumor heterogeneity.

### Prediction of primary site using IHC and special stains

The EPs were blinded to the clinical history and tissue of origin for the samples. They received only the patient’s sex and gross sample description for each case, including biopsy site, given in Table 2. Each EP first reviewed the H&E image, recorded an Initial diagnosis (Stage 1) with a level of confidence, and ordered the first round of stains from the panel in Table 1. The digitized images for these stains were provided after two working days. The EP reviewed the stain images, recorded an Intermediate diagnosis (Stage 2) with a level of confidence, and ordered the second and final round of stains from the panel in Table 1. As before, the digitized images for these stains were provided after two working days. The EP reviewed the stain images and recorded the Final diagnosis (Stage 3) with a level of confidence. The EP had the opportunity to provide a final diagnosis at any of the three stages: after the first review of the H&E image, after the first round of stains, or after the second round of stains. If the EP chose to deliver a final diagnosis at Stage 1 or Stage 2, the EP was prompted by the software to verify that this was intentional. Once the final diagnosis was provided, the EP received a system-generated e-mail with a link to the case. The EP had the option to alter the final diagnosis within 24 hours of receiving this email. After 24 hours, the case was considered closed for that EP, and no further change could be made. When two or more EP ordered the same stain, the same stain image was provided to the second ordering EP with the standard two working day delay, to ensure that no participant would be able to infer that the stain had been requested by another. The rationale for this was to ensure that the digital pathology and web interface did not provide indirect "cross talk" between EPs, i.e. to eliminate inadvertent clues on stains that were ordered by others which might allow an individual EP to infer the direction another EPs investigation was following.

**Table 2:** Specimen information

**Case**	Gender *, **	**Primary site**	**Biopsy site**	Description*
1	Female	Ovary	Omentum	This specimen received labeled with patient’s name are two, pink-yellow, lobulated omental tissue fragments measuring 14.5 × 7.0 × 1.0 cm in aggregate. Sectioning omentum reveals yellow lobulated adipose tissue marked by multiple, firm, yellow-white nodule varying in size from 0.5 × 0.5 × 0.5 cm. up to 2.0 × 1.5 × 1.0 cm. Some of these nodules show focal hemorrhage and necrosis. Representative sections are submitted in cassette. Two pieces.
2	Male	Colorectal	Liver	The specimen consists of a 360 gram, 16.5 × 7.0 × 7.0 cm segment of liver. The superior portion of the liver has been inked yellow, and the resection margin has been inked black. The liver has a pink-tan appearance with adhesions. Previous sectioning revealed a 2.5 × 2.5 cm white-tan nodule located approximately 6.0 cm from the superior portion of the liver and comes to within 1.0 cm from the resection margin. Located approximately 1.5 cm inferior to the first nodule are 2 additional white-tan, circumscribed nodules. These measure 0.2 cm and 1.3 cm in greatest dimension. The larger nodule comes to within 0.5 cm from the resection margin, and the smaller nodule comes to within 2.2 cm from the resection margin. The remaining liver has a green-brown appearance, and no additional areas of interest are identified. Second and third nodules are submitted in one cassette, 1 piece.
3	Male	Colorectal	Brain	Specimen received labeled with right frontal brain tumor. Received is a 3.0 × 2.1 × 0.3 cm aggregate of gray-tan, friable tissue fragments. Tea bag. 1 cassette, all.
4	Female	Breast	Lymph Node	This specimen received labeled with patient’s name. Contains 1 possible lymph node, 1 piece.
5	Male	Kidney	Brain	The specimen consists of a previously sectioned portion of red-gray brain tissue which upon reconstruction measures 4.0 × 3.1 × 2.0 cm. There is a 3.5 × 2.0 × 1.1 cm ill-defined yellow-gray mass which comes to within 0.3 cm of the margin. The margin has been inked black. The remaining cut surface is gray-pink, and no additional masses or areas of interest are grossly identified. Representative tissue is submitted in 1 cassette, 1 piece.
6	Female	Melanoma	Lymph Node	Possible lymph nodes, 1 cassette, multiple pieces.
7	Male	Bladder	Lymph Node	The specimen received consists of multiple fragments of fat and soft connective tissue. On sectioning, the fragments consist of possible lymph nodes ranging from 0.7 cm up to 4.3 cm in greatest dimension. The cut surfaces are solid gray-white. Possible lymph nodes, multiple pieces.
8	Male	Sarcoma	Liver	Received is a 1765 gram, 20.7 × 14.3 × 10.8 cm right lobe of liver. There is a stitch attached to the anterior aspect of the capsule. The resection margin will be inked black. On sectioning, there is a 10.4 × 6.8 cm white-tan, fleshy, friable mass. The mass is focally necrotic and in the area where the mass is necrotic, it is cystic. The mass grossly appears to come to within 1.1 cm of the resection margin. The remaining liver is red-brown and no satellite lesions or other masses are grossly identified. 1 cassette, 1 piece.
9	Male	Gastric	Omentum	The specimen consists of a 0.9 × 0.7 × 0.5 cm portion of yellow, lobulated omentum with a 1.2 cm firm, gray nodule. 1 cassette, 1 piece.
10	Male	Lung	Brain	The specimen consists of a 1.6 × 1.3 × 0.7 cm aggregate of soft tan-brown tissue fragments. Submitted in 1 cassette, multiple pieces.

* - Information given to EPs.

** - Information given for GEP testing to Pathwork Diagnostics Laboratory.

### Gene expression profiling test methodology

The specimens were processed for the Pathwork® Tissue of Origin Test at Pathwork Diagnostics Laboratory (PWDL) as described previously [14]. Unstained slides contained an identifiable tumor region that was at least 1 mm^2^ in area. To increase the percent tumor in the submitted sample, tumor tissue was microdissected (scraped) from the slides and placed into vials for RNA extraction. Total RNA was isolated using the Agencourt® FormaPure Kit (Beckman–Coulter Genomics, Beverly, MA). The total RNA was processed to prepare labeled cDNA for hybridization to Pathchip® microarrays manufactured by Affymetrix (Santa Clara, CA) with a two-cycle amplification method using the RampUP Kit (Genisphere, Hatfield, PA). A positive/negative total RNA control was run with every amplification batch. The microarrays were washed and stained using the GeneChip® Hybridization Wash and Stain kit in a GeneChip Fluidics Station FS450Dx, and scanned with a GeneChip Scanner 3000Dx (Affymetrix). Microarray data files (CEL) that passed data verification [14] were analyzed using the Tissue of Origin Test algorithm, a 2000-gene classification model which quantifies the similarity between RNA expression patterns of a study specimen and the 15 tissues on the test panel. Data were reported as Similarity Scores (SS) for each tissue, measures of the similarity of the RNA expression pattern of the specimen to the RNA expression pattern of the indicated tissue. Similarity Scores ranged from 0 (very low similarity) to 100 (very high similarity) and summed to 100 across all 15 tissues on the panel. The highest SS indicated the likely tissue of origin, with two exceptions: (i) If the highest score was less than 20, no tumor tissue type was predicted, and (ii) If the patient was male, and the highest SS was for ovarian cancer, and the second highest SS was for testicular germ cell cancer, the result was testicular germ cell cancer. For any tissue type with SS of ≤ 5, the possibility of that particular tissue type as the likely tissue of origin was ruled out. The Tissue of Origin Test result was automatically generated by the computer algorithm using only gene expression values as input. No clinical history, reference diagnosis or biopsy site information was used.

It should be noted that in clinical practice, a PWDL pathologist provides an interpretation of the test results along with a confidence level. This is based on test performance information derived from analyses of results from the clinical validation study [14], as well as histopathologic appearance, and relevant clinical information. In this study, the primary analysis was conducted using the highest SS. A PWDL pathologist recorded an interpretation of the results while blinded to the primary site, and this information was available for secondary analyses.

## Results

Ten metastatic specimens were selected from among common solid malignancies representing nine different primary sites, and four metastatic sites, as shown in Table 2. All specimens had at least 60% non-necrotic tumor content.

Five pathologists reviewed 10 cases each, for a total of 50 case reviews. The average number of stains ordered by an EP in the first round was 7.06. For 21 of 50 (42%) of the case reviews the EPs ordered a second round of stains with an average of 4.2 stains for each of these cases. From among the 442 total stains ordered by all the EPs for all the cases, an average of 22 unique stains per case was ordered by the group of five EPs. None of the EPs ordered histochemical stains other than IHC stains. The number of stains ordered for each case by individual EPs is shown in Table 3 and the twenty most commonly ordered stains are shown in Table 4. In decreasing order of frequency, the most commonly ordered stains were CK 7, CK 20, TTF-1, CDX-2, PAX-8, estrogen receptor, napsin A, PAX-2, p63, and synaptophysin. The average number of stains ordered by an evaluating pathologist (EP) per case was 8.8 (median 8, range 1 to 18).

**Table 3:** Number of stains ordered per case, per round

	**Round 1 stain orders**					**Round 2 stain orders**					**All stain orders**				**Slides used by GEP test**
	**EP 1**	**EP 2**	**EP 3**	**EP 4**	**EP 5**	**EP 1**	**EP 2**	**EP 3**	**EP 4**	**EP 5**	**Total stains per case**	**Unique stains per case**	**Range of stains ordered**	**Mean of stains ordered**	
Case 1	8	6	13	9	6	3	0	0	4	0	49	22	6-13	9.8	2
Case 2	3	4	9	6	4	0	0	0	1	0	27	10	3-9	5.4	4
Case 3	3	4	7	7	4	0	0	0	0	0	25	7	3-7	5.0	2
Case 4	9	6	12	7	7	7	3	4	6	0	61	28	7-16	12.2	2
Case 5	3	6	9	9	5	0	0	0	4	0	36	20	3-13	7.2	2
Case 6	7	6	13	9	8	9	2	5	2	0	61	31	8-18	12.2	6
Case 7	7	6	11	7	6	8	4	0	3	0	52	25	6-15	9.8	2
Case 8	10	5	11	8	5	0	2	0	7	0	48	28	5-15	9.6	2
Case 9	6	7	11	10	6	0	2	0	8	2	52	30	6-18	10.4	4
Case 10	4	5	10	8	1	0	3	0	0	0	31	15	1-10	6.2	4

**Table 4:** The numbers of EPs ordering each stain is shown by stain and case for the 20 stains most commonly ordered

**Reference diagnosis**	**Ovary**	**Colorectal**	**Colorectal**	**Breast**	**Kidney**	**Melanoma**	**Bladder**	**Sarcoma**	**Gastric**	**Lung**	**Total stains ordered**
***Case Number***	**Case 1**	**Case 2**	**Case 3**	**Case 4**	**Case 5**	**Case 6**	**Case 7**	**Case 8**	**Case 9**	**Case 10**	
***Stain Name***											
CK 7	5	5	5	4	2	4	5	2	4	4	40
CK 20	5	5	5	4	2	3	5	2	3	4	38
TTF1	3	3	4	5	3	4	5	2	3	3	35
CDX-2	2	5	5	4	1	2	5	1	4	1	30
PAX-8	3	2		4	5	3	2	2	1	2	24
Estrogen receptor	5			4	1	4	1		1		16
Napsin A	1	2	2	3	1	2	1	1		1	14
PAX-2	1	1		2	3	2	2	1	1	1	14
p63				3		1	4		2	4	14
Synaptophysin	2	1		1	2	1	1	2	2	1	13
Mammaglobin	3			3		3	1		2		12
GCDFP-15	3			4		2	2		1		12
Villin	1	2	2	1	1		1	1	2	1	12
S-100					1	4		4	1		10
WT-1	4			3		1			1		9
Pancytokeratin	1			1		2	1	2	1	1	9
PSA		1	2		1		3		2		9
CK 5/6				2		1	2		2	2	9
Hep-par				1		3		3	1		8
HMB 45					1	4		3			8

The diagnoses of primary site reached by each EP and the predictions of primary site by the GEP test are shown in Table 5. Following review of H&E slides alone, EPs reached the correct diagnosis of primary site for 21 of 50 case reviews (42% accuracy). Following review of the first round of IHC stains, the EPs reached the correct diagnosis of primary site in 31 of 50 case reviews (62% accuracy). Following the review of all IHC stains (either one round for 29 cases or two rounds of IHC for 21 cases), the EPs reached the correct diagnosis of primary site in 32 of 50 case reviews (64% accuracy). Accuracy among EPs ranged from 50% to 80%. For each EP, the accuracy increased considerably between the H&E and first round, but not between the first and second round (Table 5). In 29 out of 50 case reviews, EPs provided a final diagnosis after ordering only the first batch of stains. The average accuracy for these diagnoses was 66% while for the 21 case reviews for which two rounds of stains were ordered, the average accuracy was 62%.

**Table 5:** Diagnoses provided by evaluating pathologists and GEP test vs. the reference diagnosis

**Case No.**	**Reference Diagnosis**	**Sex**	**GEP**		**EP1**				**EP2**				**EP3**				**EP4**				**EP5**				**EP Overall, for 50 case reviews**		
			**Result**	**Highest SS**	**H&E**	**Int**	**Final**	**All**	**H&E**	**Int**	**Final**	**All**	**H&E**	**Int**	**Final**	**All**	**H&E**	**Int**	**Final**	**All**	**H&E**	**Int**	**Final**	**All**	**H&E**	**Int**	**All**
Case 1	**OV**	**F**	**OV**	97.6	BR	OV	OV	**OV**	OV	OV		**OV**	OV	OV		**OV**	OV	OV		**OV**	OV	OV	OV	**OV**	--	--	--
Case 2	**CO**	**M**	**CO**	89.9	CO	CO		**CO**	CO	CO		**CO**	CO	CO		**CO**	PA	PA		**PA**	CO	LU	LU	**LU**	--	--	--
Case 3	**CO**	**M**	**CO**	87.6	CO	CO		**CO**	CO	CO		**CO**	CO	CO		**CO**	LU	CO		**CO**	CO	CO	CO	**CO**	--	--	--
Case 4	**BR**	**F**	**BR**	97.6	LU	OV	OV	**OV**	LU	LU		**LU**	BR	BR	PA	**PA**	OV	OV	PA	**PA**	OV	OV	OV	**OV**	--	--	--
Case 5	**KI**	**M**	**KI**	86.4	KI	KI		**KI**	KI	KI		**KI**	KI	KI		**KI**	LU	KI		**KI**	KI	KI	KI	**KI**	--	--	--
Case 6	**ME**	**F**	**ME**	67.4	LY	ME	ME	**ME**	BR	BR		**BR**	LI	ME	ME	**ME**	BR	ME	ME	**ME**	LU	ME	ME	**ME**	--	--	--
Case 7	**BL**	**M**	**BL**	45.1	GC	PA	BL		LU	GA		**GA**	LU	LU	BL	**BL**	LU	BL		**BL**	BR	BL	PA	**PA**	--	--	--
Case 8	**SC**	**M**	**SC**	76.1	SC	SC		**SC**	LI	SC		**SC**	SC	SC	SC	**SC**	LI	ME		**ME**	KI	LI	LI	**LI**	--	--	--
Case 9	**GA**	**M**	**GA**	36.9	GA	GA		**GA**	SC	GA	GA	**GA**	GA	GA	GA	**GA**	PA	PA		**PA**	LY	GA	GA	**GA**	--	--	--
Case 10	**LU**	**M**	**OV**	24.4	BL	BL		**BL**	BL	BL		**BL**	BL	BL	BL	**BL**	BL	BL		**BL**	BL	BL	BL	**BL**	--	--	--
**No. incorrect**			1		5	3		**2**	6	4		**4**	3	2		**2**	**9**	**5**		**5**	6	4	5	**5**	29	19	**18**
**No. correct**			9		5	7		**8**	4	6		**6**	7	8		**8**	**1**	**5**		**5**	4	6	5	**5**	21	31	**32**
% correct			90%		50%	70%		**80%**	40%	60%		**60%**	70%	80%		**80%**	**10%**	**50%**		**50%**	40%	60%	50%	**50%**	42%	62%	**64%**

The tumor tissue is indicated by a two-letter code: bladder (BL), breast (BR), colorectal (CO), gastric (GA), testicular germ cell (GC), kidney (KI), hepatocellular (LI), non-small cell lung (LU), non-Hodgkin’s lymphoma (LY), melanoma (ME), ovarian (OV), pancreas (PA), prostate (PR), thyroid (TH) and sarcoma (SC). SS – Similarity Score, a measure of the similarity of the RNA expression pattern of the specimen to the RNA expression pattern of the indicated tissue. Similarity Scores range from 0 (very low similarity) to 100 (very high similarity) and sum to 100 across all 15 tissues on the GEP Test panel. The incorrect predictions are indicated with yellow highlight. Grey indicates no stains ordered.

The variation in the total number of stains requested by all 5 EPs per case may be indicative of relative diagnostic complexity. Using this measure, case 3 was the simplest, and all EPs provided a final diagnosis without ordering a second round of stains. One EP diagnosed the case with as few as 3 stains. The average number of stains used by all EPs was 5 (median 4, range 3–7). Case 6 was the most complex: no EP required fewer than 8 stains, and the average across all EPs was 12.2 (median 11, range 8 to 18). For both cases, all EPs and the GEP test correctly determined the primary site.

The GEP test determined the correct diagnosis in 90% (9/10) of cases. The range of highest SS was 24.4 to 97.6 (Table 5). The average number of slides used for the GEP test was three (median 2, range 2 to 6; Table 3). For cases 3 and 6, the numbers of slides used for the GEP test were 2 and 6, respectively.

## Discussion

We have devised and tested a novel approach for directly comparing a GEP test to special stain evaluation using multiple evaluating pathologists. The study design reduced subjectivity of the pathologic diagnosis and minimized the variables between the two approaches being compared. The use of a single central laboratory to perform IHC staining and digital pathology to provide the results to multiple participating pathologists eliminated the need for shipping slides from one site to another for pathologist evaluation, thus enabling more efficient study logistics and timelines. The whole slide imaging system created a digital replica of the entire content of a glass microscope slide on the computer, closely emulating traditional viewing of a slide with a conventional microscope [19]. EPs could zoom in or pan out of the web-accessible, interactive images for evaluation. The system also allowed proper control, providing access to only the stains requested by each pathologist, maintaining sample blinding and preventing indirect “cross-talk” between EPs. All the EPs were given access to images after 48 hours of ordering stains, eliminating the possibility of indirect clues to EPs regarding stains ordered by other EPs. All the slides were stained in one CLIA certified laboratory by an experienced histotechnologist using the same IHC staining instrument, thus avoiding technical variability. All the slides were digitized using only one scanner and the images were checked for quality assurance by an experienced board certified pathologist, creating uniformity in the image quality. All the images were web-accessible using a password-protected database, allowing uniformity in evaluation to all the EPs. In this study, there was no restriction on the number of stains that could be ordered. The studies in the meta-analysis [8] restricted the number of markers or IHCs in a panel to between 4 to 10, and the number of tissues of origin represented were limited to 5 to 7. In clinical practice, GEP is used as an aid to diagnosis that the pathologist uses along with all the other histopathologic and clinical information to arrive at a diagnosis. The stringent study design that we created allowed direct comparison between GEP and histopathologic evaluation.

All 5 EPs and the GEP test provided a final diagnosis on all 10 cases. Case 3 was of very low morphological difficulty and all EPs provided a final diagnosis that matched the reference diagnosis while ordering the smallest number of stains (total stains 25 among five EPs). Cases 4 and 10 were incorrectly diagnosed by all 5 EPs and warrant further discussion. The patient information provided to all the EPs and the GEP testing laboratory is shown in Table 2. For case 4, the primary site was correctly predicted by the GEP test but missed by all 5 EPs. EPs ordered a total of 61 stains; the most common being TTF1, CK7, CK20, CDX-2, PAX-2, ER and GCDFP-15. Case 4 has a somewhat confounding immunophenotype and an inconclusive appearance on H&E evaluation. In the GEP test clinical validation study [14], it was shown that there is a strong positive relationship between the Similarity Score and the probability that the TOO test prediction is correct. The highest SS generated by the GEP test was 97.6 (out of a possible 100), indicating a very high confidence in the prediction. For Case 10, neither the diagnoses reached by the EPs nor the TOO prediction matched the reference diagnosis of lung (squamous cell carcinoma). All EPs delivered a final diagnosis of bladder. Relevant IHC results for this case include positive CK7 and negative CK20, TTF-1, and uroplakin. These IHC results are fully consistent with the reference diagnosis. It is possible that the H&E appearance of this neoplasm (lack of keratinization and ribbon-like growth pattern) encouraged the EPs diagnosis of urothelial carcinoma. For Case 10, the highest SS generated was 24.4, indicating a relatively low confidence of prediction. Neither breast cancer (SS 22.9) nor non-small cell lung cancer (SS 20.8) have been excluded by the TOO test results, and would be considered possible primary sites. Bladder cancer has a very low score of 5.2, and while not formally ruled out (i.e. SS < 5), would be considered highly unlikely as a primary site.

The PWDL pathologist interpreting the GEP results favored a non-small cell lung origin, since a prediction of ovarian was implausible in males and the pattern of scores was most consistent with a non-small cell lung origin. The full value of GEP and other novel molecular evaluations in oncology will most likely be achieved by a judicious incorporation into the final pathologist consultation report [13].

The amount of tissue used by each testing method is an important consideration in evaluating tissue-based diagnostics. GEP used an average of three slides per case, whereas the EPs used an average of nine slides per EP per case. In this sample set, molecular testing required less tissue and reduced the risk of tissue depletion. For some cases (1, 4, 6, 7, 8 and 9) EPs ordered a large number of stains (average 11 slides per EP per case); whereas in cases 2, 3, 5 and 10 they ordered fewer IHC stains (average 6 slides per EP per case). The GEP test used 3 slides on average for both sets.

In this pilot study GEP performed favorably (90% accuracy) compared to histopathologic evaluation (average 64% accuracy) by 5 EPs. It is interesting to note that the average accuracy seen in this study is very similar to the average 67% accuracy reported in the previous meta-analysis [8]. In this study, there was no restriction on the number of IHCs, whereas the studies in the meta-analysis restricted the number of stains to between 4 and 10. While the sample numbers are small and do not support statistical analyses, this pilot study has established feasibility for a study with larger sample size that will be adequately powered to derive statistically significant conclusions regarding the comparative effectiveness of the two approaches.

## Conclusions

This pilot study of 10 samples found important differences in accuracy between two methods. In the 10 metastatic samples reported here, GEP identified the correct primary site more often than the IHC-guided methods used by the pathologists participating in the study. This study design will be applied to a larger set of samples to provide a statistically powered assessment of the comparative effectiveness of the GEP and IHC-guided methods.

## Abbreviations

(EP): evaluating pathologist; (FFPE): formalin-fixed paraffin-embedded; (GEP): gene expression profiling; H&E: hematoxylin-and-eosin; (IHC): immunohistochemistry; (SS): Similarity Score; (TOO): tissue of origin.

## Competing interests

CRH, AK and AME were recipients of research grants from Pathwork Diagnostics for the performance of this study. WDH and RP are employees and stock holders of Pathwork Diagnostics.

## Authors’ contributions

CRH was the Principal Investigator. CRH and AK participated in the conception, study design, sample identification, results interpretation, and correlation with clinical information and manuscript writing. AME participated in the sample identification, data management, results interpretation, and manuscript writing. RP and WDH participated in the conception, study design, conduct of the study, data management, results interpretation, and manuscript preparation. All authors read and approved the final manuscript.
